# Establishment of a special pathogen free Chinese Wuzhishan Minipigs Colony

**DOI:** 10.1186/s40781-015-0046-4

**Published:** 2015-03-01

**Authors:** Jinchun Pan, Fangui Min, Xilong Wang, Ruiai Chen, Fengguo Wang, Yuechang Deng, Shuming Luo, Jiancong Ye

**Affiliations:** Guangdong Laboratory Animals Monitoring Institute, Guangzhou, 510663 China; Guangdong Provincial Key Laboratory of Laboratory Animals, Guangzhou, 510663 China; Guangdong Dahuanong Animal Health Products Stock Co.,Ltd., Xinxing, 527439 P.R. China

**Keywords:** Chinese Wuzhishan minipigs, Specific pathogen free, Hysterectomy

## Abstract

To meet the increasing demands of specific pathogen free (SPF) minipigs in biomedical researches, 8 pregnant Chinese Wuzhishan minipigs (WZSP) sows with clear background were chosen to obtain SPF WZSP by hysterectomy. At 111 ± 2 days of the pregnancy, piglets were aseptically taken out from the sows and artificially suckled for 40 to 45 days in the positive isolators. Then, the piglets defined as F0 were transferred to barrier environment and fed with standard feeds. The original SPF colony was formed for breeding by selected piglets from F0 group of 6–8 months old. Biological characteristics of SPF WZSP were collected and further compared to those of conventional (CV) WZSP, including growth performance, reproductive performance, hematology and blood biochemistry, and major pathogens detection. As a result, 61 F0 piglets were obtained from 8 candidate sows, and 55 out of them survived. After strictly selection, 35 F0 piglets were used to form the original SPF colony, which produced 14 litters of SPF piglets defined as F1. Piglet survival rates, growth performance, and reproductive performance of SPF WZSP were similar to CV WZSP. Some hematology and blood biochemistry parameters showed significant differences between SPF and CV WZSP. Eighteen kinds of pathogens were identified to be free in F0 and F1 SPF colony by repeated pathogen detections. In conclusion, we established a satisfied SPF WZSP colony maintaining original characteristics, free of controlled diseases, and being proved to be a suitable laboratory animal.

## Background

The dog or non-human primate has been confined as the predominant non-rodent in biomedical researches for a long history [1,2]. In view of animal welfare and tenet of “3Rs”, and/or because of the similarities to human in pharmacokinetics, physiology, biochemistry and anatomy [3], swine is considered to be one of the suitable laboratory animals used in translational research, surgical models, and procedural training and is increasingly being used as a good substitution to the dog or non-human primate in preclinical toxicologic testing of pharmaceuticals [4].

As we know, Göttingen minipig, Sinclair minipig, Minnesota hormel minipig, Yucatan mini- and micro-pigs, and so on are widely used due to their detailed background information and hereditary stabilities. Though China has rich resources of minipigs, the laboratory animal work is far behind the developed countries. Chinese WZSP, grazed initially in isolated tropical areas of Hainan province [5], was firstly found and preserved in the 1980s. After that, WZSP began to be cultivated as laboratory minipigs for their small size, excellent adaptability, stable inheritance, strong disease resistance, similarities to human in physiology and anatomy [6]. Nowadays, more and more attentions have been paid to the use of WZSP [7,8].

CV minipigs, like CV rodent animals, are produced without special controls for pathogen status. For some unknown pathogens may be infected, the CV minipigs could not satisfy the growing needs for minipigs of higher health quality standards. Since the late 1950s, some developed countries have tried to cultivate SPF domestic pigs. Some SPF organizations, such as the National SPF Swine Accrediting Agency, Inc. (National SPF Agency), have developed acceptance criteria for SPF domestic pigs. In their acceptance criteria, 7 main specific growth retarding diseases and conditions are controlled, which are pneumonic lesions, turbinate atrophy, mange, lice, swine dysentery, pseudorables, and brucellosis. In Chinese national standard (CNS) of SPF swine [9], 12 pathogens should be eliminated, which are pseudorabies virus, porcine reproductive and respiratory syndrome virus, transmissible gastroenteritis of swine virus, porcine epidemic diarrhea virus, and so on. For the establishment of SPF minipigs, hysterectomy under sterile conditions is the main method [10]. In conjunction with severely controlled breeding program, the hysterectomy-obtained SPF baby pig could be routinely raised humanly.

The present study describes hysterectomy procedures, piglets breeding, and growth performances of obtained SPF F0 and F1 minipigs. Results show that our procedures and facilities can maintain the hysterectomy-obtained SPF minipigs without receiving colostrums to be free of controlled diseases.

## Methods

### Animal use approval

Animal use protocols were reviewed and approved by the Institutional Animal Care and Use Committee of Guangdong Laboratory Animal Monitoring Institute in accordance with the *Guide for the Care and Use of Laboratory Animals* [11]. Animal work was conducted using institutional operating procedures and policies in a barrier facility with approval of an oversight by the Institutional Environmental Health and Safety Office.

### Establishment of SPF colony

**Figure 1:**
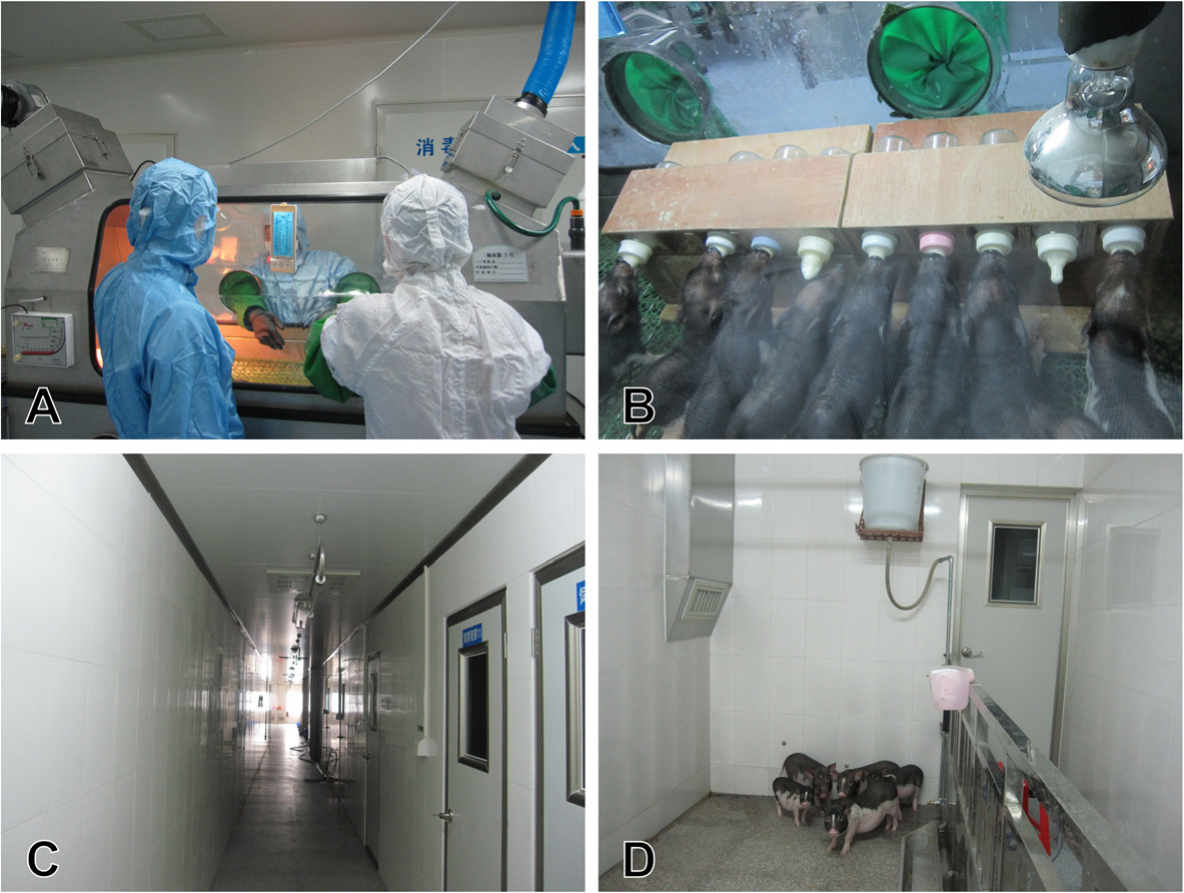
**The facility and habitats of SPF WZSP colony. A**. researchers were operating the isolator; **B**. piglets were fed with milk power by the breastfeeding frames in the isolator; **C**. the corridor of the barrier system; **D**. SPF piglets in the barrier system.

Eight adult candidate sows were strictly selected with clear background and free of porcine reproductive and respiratory syndrome virus, swine fever virus, pseudorabies, brucella bacteria, and toxoplasma. After fertilization, pregnant sows were transferred to clean delivery room at day 100 of gestation, and close observation and care followed to be performed. At 111 ± 2 days of gestation, hysterectomy procedures were carried out as described previously [12]. Feeds and water were forbidden for at least 18 h before caesarean sections of pregnant sows. Obtained piglets were bred in positive isolators with high efficiency particulate air filter for 40-45d (Figure 1A, B). During this period, piglets were fed humanly with milk powder at first and additional feeds after two weeks. Then all survival piglets were transferred to barrier environment and fed with standard feeds only. In order to establish a closed SPF colony, strong piglets should be chosen from different sows to form original SPF colony. And the minipigs were used to reproduce for generations in the established original SPF colony when they were 6–8 months old.

### Breeding environment control measures

According to the CNS, barrier and isolation environment are suited to raise SPF laboratory animals [13]. Positive isolators were used to breed suckling piglets for reducing the risk of infection, and barrier environment to breed weaned pigs for gaining more space for activities (Figure 1). The parameters of positive isolators and barrier environment were listed in Table 1, which met the demands of the CNS. The synthetic milk powder was sterilized by gamma irradiation (dosage: 20 kGy), and the complete formula feed was sterilized by autoclave sterilization (121°C, 30 min). The drinking water was sterilized by autoclave (121°C, 30 min) in the isolation environment, and the acidified water (pH = 3.0) was used in the barrier environment. Other instruments and reagent were all sterilized by physical or chemical disinfection methods before entering the barrier or isolation environment.

**Table 1:** **Parameters of positive isolators and barrier environment**

**Items**	**Units**	**Parameters of detection**	
		**Positive isolators**	**Barrier environment**
Temperatures	°C	23.5-23.8	19.4-20.1
Absolute humidity	%	64.2-66.8	67.0-68.8
Air exchange	times/h	32-41	10
Air velocity	m/s	0.14-0.18	0.14-0.15
Air pressure	Pa	100-149	10-20
Air cleanliness (≥0.5 μm)	pc/m^3^	0-1295	-
Number of dropped bacterial (Static state)	per plate	None	0-3
Intensity of illumination	lx	179-250	214-232

### Growth Performance

The minipigs were weighed routinely at 1 week interval from 0 to 4 weeks old, 4 weeks interval from 1 to 6 months old, and 8 weeks interval from 6 to 12 months old. And the results of body weight (BW) were compared with each other among SPF F0, F1 and CV WZSP.

### Reproductive Performance

Reproductive performances were routinely recorded for 14 SPF sows and 45 CV sows at birth and at weaning. And the differences between SPF and CV sows were analyzed by student’s *t*-test.

### Hematology and Blood Biochemistry

About 2.0 mL blood samples were obtained via popliteal venipuncture for clinical hematology and blood biochemistry analysis after fasting for 12 h. Haematological indices were determined by automatic hematology analyzer (Sysmex XT-2000iv), and blood biochemistry indices were determined by automatic biochemical analyzer (Hitachi 7020).

### Pathogens detection

The sera, dander and hair of animals were collected for pathogen detection. The sampled minipigs included 8 pregnant sows for cesarean, 9 SPF F0 minipigs (4 males and 5 females), 2–3 months of age, 10 SPF F0 minipigs (5 males and 5 females), 14–15 months of age, and 20 SPF F1 minipigs (10 males and 10 females), 2–3 months of age. The methods for pathogens detection are described in Table 2. All detections were performed by commercial kits according to manufacturer’s instructions. The kits of PRV-gE, PRV-Ab, and PRRSV-Ab were purchased from Beijing IDEXX Yuanheng Laboratories Co.,Ltd. PPV, HPS and JEV kits were purchased from Wuhan Keqian Animal Biological Products Co.,Ltd. CSFV-Ab and CSFV-Ag kits were purchased from Korea JBT INC. Toxoplasma and chlamydia kits were purchased from Lanzhou veterinary research institute of Chinese academy of agricultural sciences. PCV2-Ab kit, FMDV-Ab, Brucella and IV kits were purchased from Ringpu (Baoding) biological pharmaceutical Co.,Ltd., Beijing jinnuo baxter technology Co., Ltd., and China institute of veterinary drugs control and Beijing wantai biological pharmacy enterprise Co., Ltd respectively. TGEV, PEDV, TPm, MH and APP kits were from South China Agricultural University.

**Table 2:** **Pathogens and detection methods**

**Detection methods**	**Pathogens**
ELISA	PRV, FMDV, PRRSV , CSFV, TGEV, PEDV, TPm, MH, APP
Latex agglutination test	JEV, PPV
Plate agglutination test	Brucella, HPS
Indirect hemagglutination test	Toxoplasma, Chlamydia
Hemagglutination inhibition test	IV
Micro-examination	Eperythrozoon, Ectoparasites

Note: PRRSV: porcine reproductive and respiratory syndrome virus; CSFV: classical swine fever virus; FMDV: foot and mouth disease virus; PRV: pseudorabies virus; JEV: Japanese encephalitis virus; PPV: porcine parvovirus; HPS: haemophilus parasuis; TGEV: transmissible gastroenteritis of swine virus; PEDV: porcine epidemic diarrhea virus; TPm: toxigenic pasteurella multocida; MH: mycoplasma hyopneumoniae; APP: actinobacillus pleuropneumoniae; IV: influenza virus.

### Statistical analysis

All numerical parameters were expressed as mean ± SD. The data were statistically assessed by Student’s *t*-test. Significance was judged at the *p* < 0.05 level.

## Results

### Hysterectomy and F0 piglet breeding

Sixty-one piglets were obtained from 8 candidate sows after aseptic surgery and hysterectomy, 55 out of them survived after cardio pulmonary resuscitation, and 49 piglets were survival at weaning. During the artificial lactating period, a breastfeeding frame was designed to reduce manpower (Figure 1B). After weaning, piglets were fed with autoclaved feeds and acidified water which were determined to be sterile repeatedly. The original SPF colony was formed by 35 survival F0 piglets from 8 candidate sows. In the end, we gained 14 litters of SPF F1 minipigs by reproducing in the original SPF colony.

### Growth Performance

**Figure 2:**
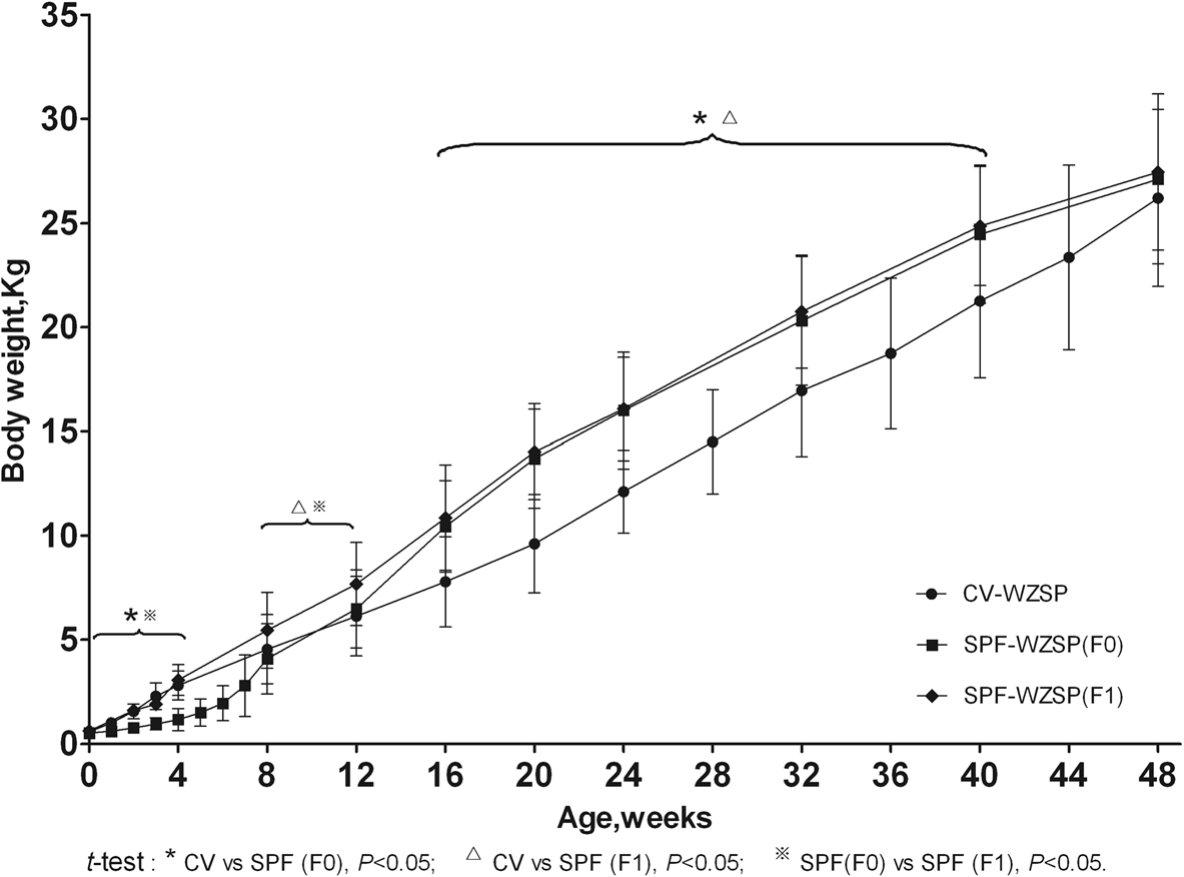
**Growth curve for BW of different ages.** The BW of SPF F0 minipigs showed a slower increase before weaning and a sharper increase after weaning compared to that of CV minipigs (*P* < 0.05). The BW of SPF F1 minipigs showed similar increase before weaning and higher increase after weaning compared to that of CV minipigs.

Growth curve of BW was shown in Figure 2. As a result, the dynamic changes of BW in SPF F0 minipigs were different from CV minipigs demonstrating a slower increase before weaning and a sharper increase after weaning (*p* < 0.05). However, SPF F1 minipigs showed similar increase to CV minipigs before weaning (*p* > 0.05), higher increase to CV minipigs (*p* < 0.05) and similar increase to SPF F0 minipigs (*p* > 0.05) after weaning.

### Reproductive Performance

Reproductive performances recorded for 8 candidate sows, 14 SPF colony sows and 45 CV sows were shown in Table 3 and compared with each other. Reproductive behavior parameters of SPF WZSP, including estrus cycle, estrus duration, gestation period, were similar to CV WZSP (*p* > 0.05) [14]. The average survival rate of piglets at weaning of 8 candidate sows (80.3%) was lower than that of SPF F0 sows (82.2%) and CV sows (85.8%), but there were no statistical differences (*p* > 0.05). In a word, there were no significant differences in litter performances among the three kinds of sows analyzed by student’s *t*-test (*p* > 0.05).

**Table 3:** **Reproductive performances of SPF and CV WZSP sows**

**Reproductive performances**	**Candidate sows(n = 8)**	**SPF colony(n = 14)**	**Outbred colony(n = 45)**
Total litter sizes	7.63 ± 1.30	6.46 ± 1.98	6.35 ± 1.53
Survival numbers at birth	6.88 ± 1.55*	5.46 ± 1.94	6.23 ± 1.53
Survival numbers at weaning	6.13 ± 1.13	5.31 ± 1.75	5.45 ± 1.95
Estrus cycle	-	21.22 ± 0.88	21.16 ± 0.75
Estrus duration	-	3.98 ± 0.42	4.18 ± 0.31
Gestation period	-	113.55 ± 2.50	113.0 ± 2.33

Note: *Survival numbers after hysterectomy.

### Hematology and Blood Biochemistry

Hematology and blood biochemistry indices play an important role in evaluation of minipigs’ homeostasis and disease diagnosis. Tables 4 and 5 present the background data of hematology and blood biochemistry for SPF WZSP and comparison of SPF WZSP with CV WZSP.

**Table 4:** **Determination of hematological parameters**

**Parameters**	**Abbrv.**	**Unit**	**SPF WZSPs (n = 49)**	**CV WZSPs (n = 44)**
White blood cells	WBC	1 × 10^9^ /L	15.38 ± 5.61	16.97 ± 5.48
Red blood cells	RBC	1 × 10^12^ /L	8.15 ± 1.52	8.74 ± 1.86
Haemoglobin	HGB	g/L	134.82 ± 25.68*	155.98 ± 28.78
hematocrit	HCT	%	47.49 ± 9.81*	51.79 ± 9.57
Mean cell volume	MCV	fL	58.42 ± 7.78	59.79 ± 5.88
Mean cell hemoglobin	MCH	pg	16.58 ± 1.35*	18.00 ± 1.70
Mean cell hemoglobin concentration	MCHC	g/L	286.73 ± 30.56*	301.50 ± 12.23
Total Platelet count	PLT	1 × 10^9^ /L	404.17 ± 221.03	339.43 ± 109.80
Platelet distribution width-SD	RDW-SD	fL	43.57 ± 8.87	21.98 ± 2.69
Platelet distribution width-CV	RDW-CV	%	23.04 ± 2.82	14.13 ± 2.47
Neutrophils	NEUT	1 × 10^9^ /L	2.60 ± 1.63*	6.53 ± 2.63
Lymphocytes	LYMPH	1 × 10^9^ /L	10.49 ± 6.4	9.30 ± 4.73
Monocytes	MONO	1 × 10^9^ /L	0.80 ± 0.32*	0.60 ± 0.25
Eosinophils	EOS	1 × 10^9^ /L	0.30 ± 0.18	0.35 ± 0.22
Basophils	BASO	1 × 10^9^ /L	0.05 ± 0.04*	0.19 ± 0.15

Note: Results of *t-*test. Comparison between SPF and CV WZSP. *P < 0.05.

**Table 5:** **Determination of the haemal biochemical parameters**

**Parameters**	**Abbrv.**	**Unit**	**SPF WZSPs (n = 14)**	**CV WZSPs (n = 37)**
Alanine aminotransferase	ALT	U/L	58.71 ± 9.89*	78.61 ± 21.37
Aspartate aminotransferase	AST	U/L	63.29 ± 16.65	62.26 ± 29.60
Alkaline phosphatase	ALP	U/L	135.00 ± 24.39*	243.89 ± 144.99
Total protein	TP	g/L	84.86 ± 10.85	83.81 ± 9.01
Albumin	ALB	g/L	51.73 ± 6.78*	33.88 ± 5.30
Globulin	GLB	g/L	33.15 ± 5.90*	49.99 ± 8.67
Blood urea nitrogen	BUN	mmol/L	3.44 ± 1.12	5.06 ± 3.74
Creatinine	CREA	μmol/L	107.43 ± 24.99*	136.18 ± 41.78
Glucose	GLU	mmol/L	5.25 ± 2.38	5.71 ± 2.39
Cholesterol	CHOL	mmol/L	2.72 ± 0.77	2.39 ± 0.68
Total bilirubin	TBILI	μmol/L	0.51 ± 0.36	0.46 ± 0.56
Triglycerides	TG	mmol/L	0.60 ± 0.30	0.59 ± 0.24

Note: Results of *t-*test. Comparison between SPF and CV WZSPs. *P < 0.05.

In 15 hematological parameters, HGB, HCT, MCH, MCHC, NEUT, MONO, and BASO showed significant differences between SPF and CV WZSP (*p* < 0.05). Five indices out of 12 blood biochemical parameters showed significant differences between SPF and CV WZSP (*p* < 0.05), which were ALT, ALP, ALB, GLB, and CREA.

### Pathogen detection

Pathogen detection results of CV candidate sows and SPF WZSP were listed in Table 6. For 8 candidate sows, 3 kinds of pathogens were detected out with detection rate of 50% (4/8), 100% (8/8) and 100% (8/8) for HPS, IV and PPV. However, 18 kinds of detected pathogens were successfully forbidden in F0 piglets by hysterectomy procedures. The F1 piglets were keeping free of those pathogens by repeated detection, indicating a satisfy SPF minipigs colony and effective SPF control system.

**Table 6:** **Pathogen detection of SPF WZSP**

**Pathogen**	**Positive number**		
	**CV candidate sows (n = 8)**	**F0 minipigs (n = 19)**	**F1 minipigs (n = 20)**
PRV	0	0	0
FMDV	0	0	0
JEV	0	0	0
Brucella	0	0	0
HPS	4	0	-
Chlamydia	0	0	0
Eperythrozoon	0	0	0
CSFV	0	0	0
PRRSV	0	0	0
Toxoplasma	0	0	0
Ectoparasites	0	0	0
IV*	8	0	0
TGEV	-	0	0
PEDV	-	0	0
TPm	-	0	0
MH	-	0	0
APP	-	0	0
PPV	8	0	0

Note: *Twelve subtypes of IV were detected, which were CA07(H1N1-pdm), Sw1304(H1N2-CS), Sw1110(H1N2-TR), Sw72(H1N1-EA), SwNS2788(H1N1-re), SwNS2811(H3N2-re), Brisb10(H3N2), ST55(H3N2), G1(H9N2), Y280(H9N2), NT155(H9N2), NT449(G1).

## Discussion

With the development of medical and biological science, the minipigs have subsequently gained regulatory acceptance in pharmacology, toxicity and basic researches [4]. Recent evidence suggests a increasing demand of high-quality minipigs, which can reduce the interference of some inapparent pathogens [15]. However, ensuring an adequate supply of high-quality minipigs may become a main challenge. Addressing these concerns, we performed the present study.

To obtain F0 SPF minipigs, 8 candidate sows, free of CSFV, PRRSV, PRV, FMDV, brucella, and toxoplasma that might influence reproductive performance of sows [16], were selected for hysterectomy procedures. At 111 ± 2 days of gestation, hysterectomy procedures were performed with a survival rate of 90% (55/61) for F0 piglets. After weaning, 35 F0 minipigs were selected out to construct a core group, which was used to reproduce. Fourteen litters of F1 minipigs were obtained from F0 group. The F0 and F1 groups were our objective for this study.

Then, serial biological indices of SPF WZSP were collected periodically by experienced technicians. Results showed that the dynamic changes of BW in SPF F0 minipigs were a little different from CV minipigs demonstrating a slower increase before weaning and a sharper increase after weaning. Previous reports revealed that caesarian section and artificial breastfeeding could reduce growth rates of the neonatal pig [17], which were consistent with the growth curve of SPF F0 piglets before weaning. However, in our research, comfortable environments and free of special pathogens significantly enhanced the growth rate of both SPF F0 and F1 groups after weaning by a comparison between SPF and CV minipigs (*t*-test, *P* < 0.05). When compared to Göettingen minipig, a famous and widely used minipig [18], the growth curve of SPF WZSP highly resembled that of Göettingen minipig.

There were no significant differences in reproductive behavior parameters between SPF and CV WZSP. No significant differences between SPF and CV sows in litter performances were observed too, including the total litter sizes at birth, survival numbers at birth and at weaning. Those results indicated that the SPF colony maintained intrinsic productive performances.

Hematology and blood biochemistry are crucial physiological parameters reflecting health status of body and important evaluation indexes in the research of biological sciences [19,20]. In 15 hematological parameters and 12 blood biochemical parameters, 7 and 5 of them showed significant differences between SPF and CV WZSP respectively. The major differences might be caused by different breeding procedures, feeds and microorganisms carrying in WZSP.

In our work, the candidate sows were free of at least 6 kinds of pathogens, which make it easier to eliminate pathogens in F0 generation. The SPF WZSP were further identified to be free of 17 pathogens and 12 subtypes of IV. Our results proved that some certain kinds of pathogens could be completely eliminated by hysterectomy, such as HPS, IV and PPV, in which PPV may transmit via transplacental route. Those SPF minipigs have been used to study the biological characteristics of H7N9 influenza virus that newly occurred in 2013 by Professor Guan of Hong Kong University and proved to be a suitable animal model [21].

## Conclusions

We succeeded to establish an ideal SPF WZSP colony which was in clean state of 18 main pathogens as well as maintained original characteristics. The SPF WZSP could be used for the study of pathogenic microorganisms, xenograft research, immunology and oncology research, and pork industry. The SPF WZSP should also afford new opportunities for other biomedical researches. In a word, the SPF WZSP have a bright practical prospect.
